# Beta-Lapachone Attenuates BMSC-Mediated Neuroblastoma Malignant Transformation by Inhibiting Gal-3/Gal-3BP/IL6 Axis

**DOI:** 10.3389/fphar.2021.766909

**Published:** 2021-11-01

**Authors:** Yang Zhou, Hui Yan, Qiang Zhou, Ruiling Feng, Penggao Wang, Fang Yang, Yaodong Zhang, Ziqiao Yuan, Bo Zhai

**Affiliations:** ^1^ Children’s Hospital Affiliated to Zhengzhou University, Henan Children’s Hospital, Zhengzhou Children’s Hospital, Zhengzhou University, Zhengzhou, China; ^2^ Department of Cardiothoracic Surgery, Children’s Hospital Affiliated to Zhengzhou University, Henan Children’s Hospital, Zhengzhou Children’s Hospital, Zhengzhou, China; ^3^ Department of Pathology, Children’s Hospital Affiliated to Zhengzhou University, Henan Children’s Hospital, Zhengzhou Children’s Hospital, Zhengzhou, China; ^4^ School of Pharmaceutical Sciences, Zhengzhou University, Zhengzhou, China

**Keywords:** neuroblastoma, tumor microenvironment, IL6, BMSC, beta-lapachone

## Abstract

The inflammatory factor IL6 secreted by bone marrow mesenchymal stem cells (BMSCs) in the tumor microenvironment (TME) facilitates the survival and therapeutic resistance of neuroblastoma (NB). Here, we found that IL6 expression in primary tumor tissues or bone marrow (BM) metastases was closely associated with the disease risk and prognosis of NB patients. IL6 secretion from immortalized BMSC (iBMSC) was directly regulated by NB cells and is involved in promoting the proliferation and metastasis of NB cells. Beta-Lapachone (ARQ-501, LPC), an *ortho*-naphthoquinone natural product, significantly prevented the iBMSC-induced malignant transformation effect on NB cells through suppressing the expression and secretion of IL6 from iBMSC *in vitro* and *in vivo*. Mechanistically, LPC disrupted the crosstalk between NB cells and iBMSC in an NQO1-dependent manner through blocking the Gal-3/Gal-3BP/IL6 axis. Our results reveal the effect of iBMSC-derived IL6 on TME-induced malignant transformation of NB cells, and provide theoretical basis for the clinical application of LPC as a potential IL6 inhibitor in high-risk refractory NB patients.

## Introduction

Neuroblastoma (NB) originates from abnormally differentiated neural crest precursors and is the most common and lethal embryonic solid tumor in childhood (Rickman et al., 2018). Although surgery, chemoradiotherapy, myeloablating and targeted therapy have greatly improved the survival of NB patients, the therapeutic effect on high-risk NB is still unsatisfactory (Bosse and Maris, 2016). With a comprehensive understanding of tumor characteristics, therapies directed against cancer cells themselves have been expanded to attack the complex inflammatory tumor microenvironment (TME) (Blavier et al., 2020). Inflammatory TME facilitates the malignant proliferation, drug resistance, immune escape and metastasis of NB cells (Blavier et al., 2020; Joshi, 2020). Interfering with the crosstalk between inflammatory TME and tumor cells will provide a potential breakthrough for NB therapy.

Multiple inflammatory mediators secreted by stromal cells in TME are the important part of their interaction with NB tumors. Among them, IL6 derived from BMSCs plays a critical role in the growth and survival of NB cells and the formation of therapy-resistant TME (Egler et al., 2008; Ara et al., 2009; Ara et al., 2013). Increased IL6 levels in peripheral blood and BM of NB patients reflect the characteristics of high-risk disease and poor prognosis (Egler et al., 2008; Ara et al., 2009; Ara et al., 2013). In addition, rs1800795 and rs8192284 single nucleotide polymorphism of IL6 in NB samples was found to be an independent prognostic marker for high-risk NB (Lagmay et al., 2009; Totaro et al., 2013; Zhao et al., 2018). Therefore, IL6 has become one of the most concerned inflammatory factors in the research field of NB. In turn, cytokines secreted by NB cells may also contribute to the production of inflammatory factors in BMSC (Ara et al., 2013; Nakata et al., 2017). Studies have found that galectin-3-binding protein (Gal-3BP) secreted from NB cells upregulates the secretion and expression of IL6 in BMSCs by binding to the receptor protein galectin-3 (Gal-3) on BMSC (Fukaya et al., 2008; Silverman et al., 2012).

Natural products extracted from plants, animals or microorganisms have played an important role in the struggle against diseases for centuries (Wang et al., 2020). Beta-Lapachone (ARQ-501, LPC), an *ortho*-naphthoquinone natural product isolated from the lapacho tree (*Tabebuia avellanedae*), has attracted extensive attention due to its multiple pharmacological activities of anti-tumor, anti-bacterial, anti-malarial, ant-viral and anti-inflammatory activities (Guiraud et al., 1994; Gupta et al., 2002; Mokarizadeh et al., 2020; Gong et al., 2021). As the activatable substrate of NQO1, LPC can produce a large amount of reactive oxygen species after being reduced, which exert powerful anti-tumor activity in many cancers, such as lung cancer, colon cancer and pancreatic cancer (Kim et al., 2005; Bey et al., 2007; Gong et al., 2021). In addition, LPC also exhibits anti-inflammatory effects in many inflammatory diseases such as acute pancreatitis, Alzheimer’s disease, multiple sclerosis, arthritis (Shen et al., 2017; Mokarizadeh et al., 2020). Although LPC exerts pharmacological activities in a variety of cancers and inflammatory diseases, its effect on the crosstalk between NB cells and the inflammatory TME has not been investigated.

In the current study, we found that high expression of IL6 in primary tumor tissues or BM metastases was closely associated with higher risk disease grade and poorer survival of patients based on the results of IHC analysis of NB specimens and bioinformatic analysis of NB transcriptome data from public databases (GSE3960). Immortalized BMSC (iBMSC) secreted-IL6 were significantly upregulated when iBMSC were co-cultured with NB cells or stimulated by conditioned medium (CM) derived from NB cells. IL6 derived from iBMSC promoted the proliferation and metastasis of IL6R-expressing NB cells in a paracrine manner, but had no obvious effect on IL6R-deficient NB cells. Interestingly, LPC significantly prevented iBMSC-induced promotion of proliferation and migration of NB cells through reducing the IL6 stimulatory activity of NB cells on iBMSC *in vitro* and *in vivo*. Mechanistically, LPC downregulates the Gal-3/Gal-3BP/IL6 axis between NB cells and iBMSC in an NQO1-dependent manner, thereby inhibiting the tumor-promoting effect of iBMSC-derived IL6 on NB cells.

## Results

### IL6 Expression is Correlated With the Clinicopathological Characteristics of Neuroblastoma Patients

**FIGURE 1:**
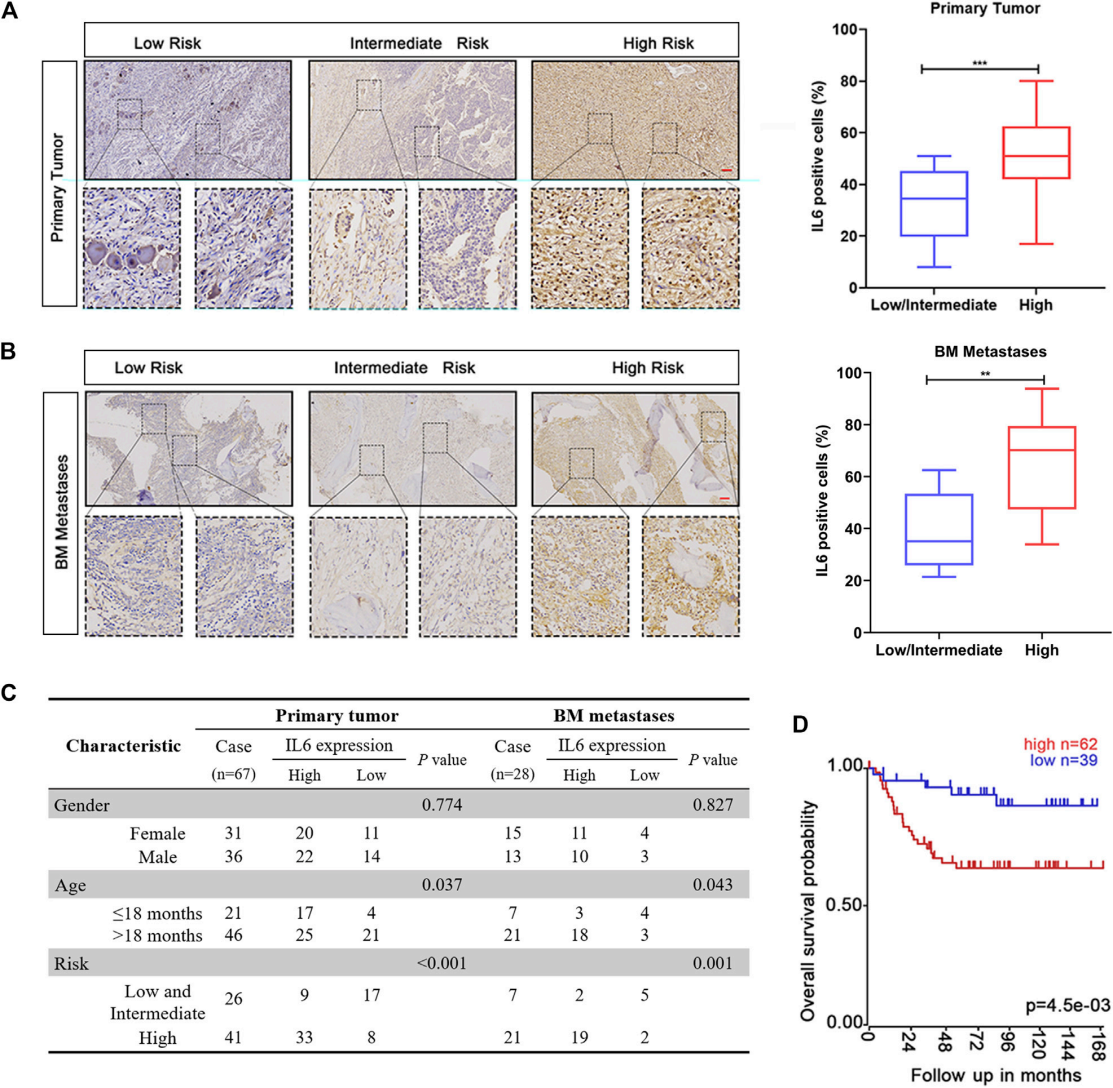
The expression of IL6 is related to the clinical factors and prognosis of NB patients. **(A,B)** IHC staining of IL6 was detected in NB primary tissues and BM metastases. Scale bars, 100 μm ***p < 0.001 compared with Low/intermediate risk patients. **(C)** The protein expression of IL6 in NB primary tissues and BM metastases. **(D)** Correlation between patients’ clinical characteristics and IL6 protein expression. *p* values were calculated using Pearson’s χ2 test and Fisher’s exact test.

Studies have confirmed that increased IL6 levels in peripheral blood and BM of NB patients reflect the characteristics of high-risk disease and poor prognosis (Egler et al., 2008; Ara et al., 2009; Ara et al., 2013). However, the expression of IL6 in NB primary tissues or BM metastases and its indicative significance for reflecting the disease progression of patients have not been fully clarified. To clarify the expression of IL6 in NB, we collected a series of NB samples with different risk grades, including 67 primary NB samples and 28 BM metastasis samples. As shown in Figures 1A,B, IL6 IHC staining showed that the expression of IL6 in both two types of tumor samples from high-risk patients was significantly higher than those of the corresponding low and intermediate risk populations. We also noticed that the IL6 expression is higher in tumor tissues of children older than 18 months (Figure 1C). In addition, the expression level of IL6 in BM metastases was significantly higher than that in NB primary tissues (Supplementary Figure S1). Moreover, Kaplan-Meier survival analysis of the GSE3960 cohort showed that high IL6 expression was associated with low overall survival, while low IL6 expression was associated with better prognosis (https://r2platform.com) (Figure 1D). The above results revealed that the expression level of IL6 in tumor tissues may be an indicator for predicting the risk classification and prognosis of NB patients.

### Neuroblastoma Cells Mediated iBMSC-Derived IL6 Secretion and Induced the Activation of STAT3 and ERK1/2 Signaling Pathways in IL6R-Expressing Neuroblastoma Cells

**FIGURE 2:**
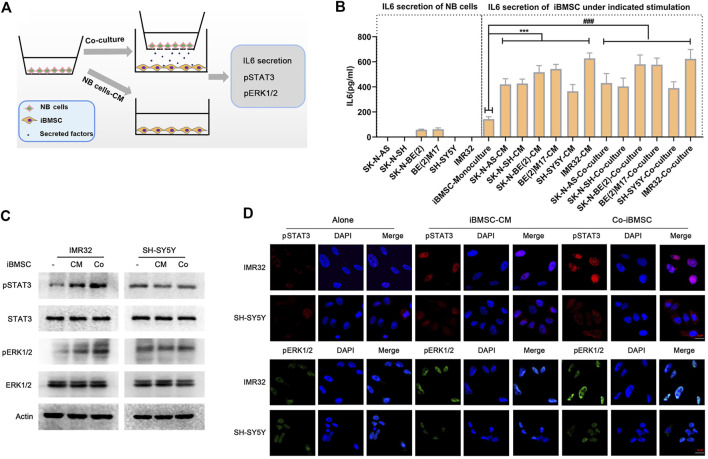
NB cells stimulated IL6 secretion from bone marrow mesenchymal stem cells. **(A)** Schematic diagram of iBMSC stimulated with NB cells CM or co-cultured with NB cells. That is, iBMSC were cultured with CM derived from 6 NB cells or co-cultured with NB cells for 48 h. **(B)** The content of IL6 was detected by ELISA in the supernatant of the corresponding culture system. NB cells were co-culture with iBMSC or stimulated by NB cells-priming iBMSC CM. **(C)** The protein expression levels of pSTAT3, STAT3, pERK1/2 and ERK1/2 in NB cells were detected by WB. **(D)** The phosphorylation levels and localization of pSTAT3 and pERK1/2 in NB cells were detected by immunofluorescence. Data represents three independent experiments and is presented as mean ± SD (***p < 0.001 vs. iBMSC-Monoculture).

Studies have found that IL6 in the NB microenvironment is not derived from the tumor cells themselves, but is secreted by surrounding stromal cells, among which BMSC is closely involved as an important TME factor (Ara et al., 2009). To explore the role of IL6 in NB cells-BMSC interaction, iBMSC was cultured alone or co-cultured with NB cells or stimulated with NB cells-derived CM, and the IL6 secretion in the medium supernatant was subsequently detected (Figure 2A). We found that NB cells hardly secreted IL6, and iBMSC secreted a small amount of IL6, which was greatly up-regulated after being co-cultured with NB cells or stimulated with NB cells CM (Figure 2B). This finding is almost consistent with what previous studies have reported (Ara et al., 2009). Subsequently, we examined IL6R expression in six neuroblastoma cell lines in our laboratory and found that IL6R was expressed in most NB cell lines except SH-SY5Y cell (Supplementary Figure S2). To further verify the effect of IL6 secreted by iBMSC on NB cells, we monitored the activation of the IL6 classical downstream signals STAT3 and ERK1/2. As shown in Figures 2C,D, co-culture with iBMSC or stimulated by NB cells-priming iBMSC CM resulted in an up-regulation of phosphorylated STAT3 and ERK1/2 in NB cells with IL6R-expressing, but not in IL6R-deficit NB cell (SH-SY5Y). Our and previous results suggest that IL6 is a potential mediator of crosstalk between tumor cells and BMSC in NB.

### IL6 Secreted by Bone Marrow Mesenchymal Stem Cell Is Essential for Maintaining the Malignant Phenotype of Neuroblastoma

**FIGURE 3:**
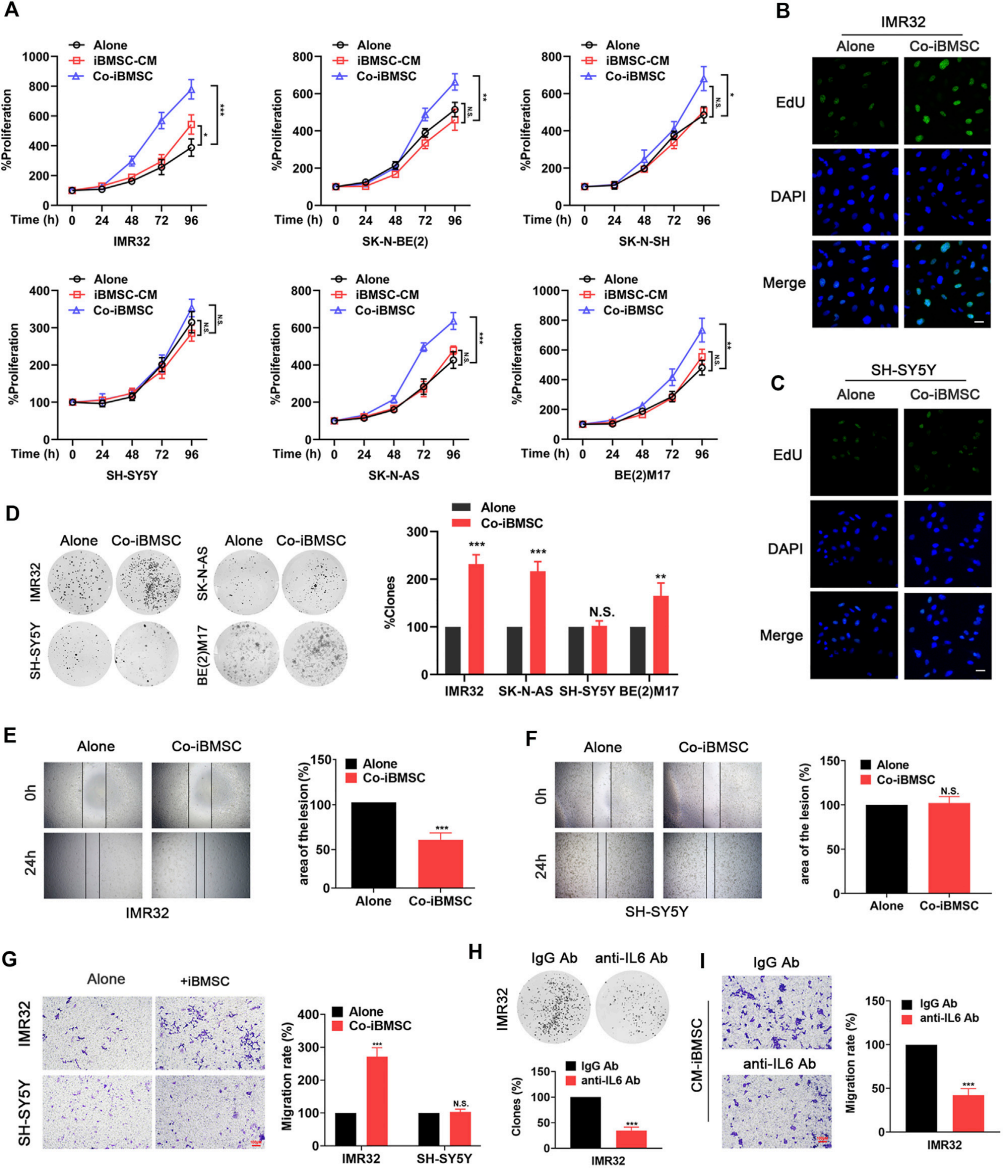
IL6 secreted by iBMSCs is an important inflammatory factor that facilities the proliferation and migration of NB cells. The NB cells were cultured alone or co-cultured with iBMSC or cultured with iBMSC derived CM. **(A)** The viability of NB cells was detected by MTT assay. (*p < 0.05, **p < 0.01 and ***p < 0.001 vs. iBMSC-Alone) **(B, C)** The proliferation of IMR32 and SH-SY5Y cells was measured by EdU incorporation method. **(D)** Clone formation analysis was performed after 2 weeks of culture. (**p < 0.01 and ***p < 0.001 vs iBMSC-Alone) **(E,F)** Wound-healing assay was performed to reflect the migration ability of IMR32 and SHSY5Y in co-culture with and without iBMSC. (***p < 0.001 vs iBMSC-Alone) **(G)** Transwell migration assay of the IMR32 and SHSY5Y in co-culture with and without BMSC. Left: A crystal violet stain of migrating cells. Right: Rate of cells migrating through the membrane. Scale bar, 100 μm. IMR32 was cultured with IL6 immune-deprived CM from priming iBMSC. (***p < 0.001 vs. iBMSC-Alone) **(H)** Colony-forming assay. **(I)** Migration assay. (***p < 0.001 vs. IgG Ab) Data represents three independent experiments and is presented as mean ± SD.

Exposure to IL6 derived from BMSC was found to facilitate NB cells survival and chemotherapy resistance (Ara et al., 2009; Ara et al., 2013). Here, we investigate other aspects of the malignant phenotype conferred by iBMSC on NB cells, including cell proliferation and migration characteristics. As shown in Figure 3A, co-culture of BMSC significantly promoted the growth of IL6R-expressing NB cells, but had no significant effect on IL6R-deficit NB cell. It is worth noting that the CM derived from iBMSC without NB cell priming had little effect on the phenotypic transformation of NB cells. Similarly, compared with cultured alone, co-culture with iBMSC significantly improved the number of clone formation, cell viability, EdU staining positive rate and metastatic characteristics of IL6R-expressing IMR32 cell, but had no significant effect on IL6R-deficit SH-SY5Y cell (Figures 3B–G). Interestingly, IL6 blockade with humanized anti-IL6 Abs administration in CM derived from NB cells-priming iBMSC significantly reversed promotion effects of iBMSC on the cell proliferation and migration in IMR32 and SK-N-AS (Figures 3H,I). The above results revealed that IL6 may be an important inflammatory mediator of the interaction between BMSC and NB cells and plays a key role in shaping the malignant phenotype of NB.

### Beta-Lapachone Prevents the Protumorigenic Function of Immortalized BMSC Through Affecting Neuroblastoma Cells-Stimulated IL6 Secretion by Bone Marrow Mesenchymal Stem Cell

**FIGURE 4:**
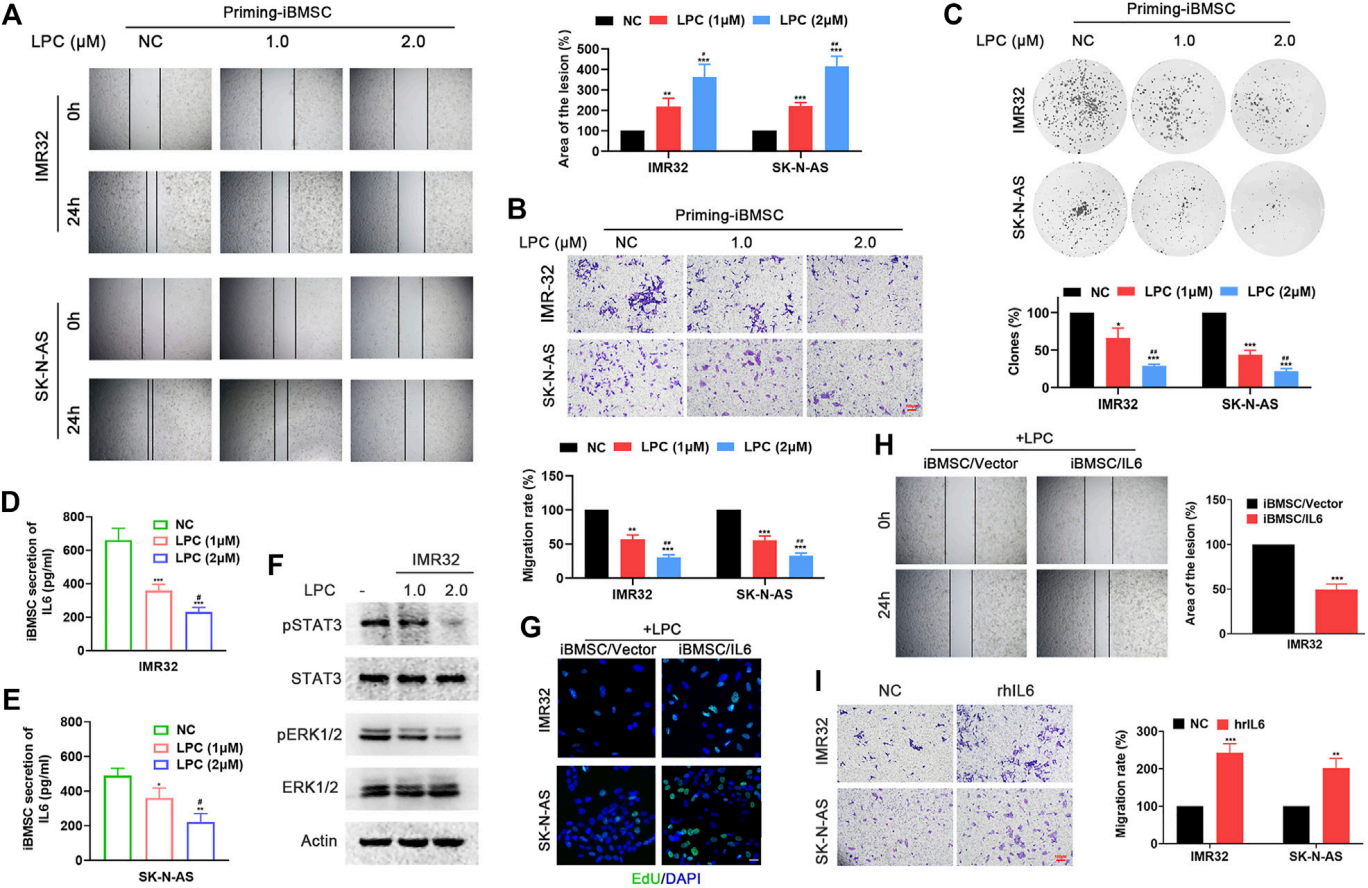
LPC hinders the tumorigenic effect of iBMSC by inhibiting the secretion of IL6 in BMSC mediated by NB cells. NB cells pretreated with different doses of LPC were continued to be cultured in fresh medium for another 48, then the supernatant was extracted and as LPC pretreatment condition medium (LPC-CM). LPC-CM was used to prime the iBMSC, which were then co-cultured with non-LPC pretreated NB cells for a new round of culture. [**p < 0.01 and ***p < 0.001 vs. NC; *#p* < 0.01, *##p* < 0.01 and *###p* < 0.001 vs. LPC (1 μM)] **(A)** Wound-healing assay. **(B)** Transwell migration assay. **(C)** Colony formation assay. **(D,E)** IL6 secretion in the supernatant of iBMSC cultured with LPC-CM was detected by ELISA. **(F)** WB method was performed to detect the phosphorylation levels of STAT3 and ERK1/2 in IMR32 cultured with CM from LPC-CM stimulated-iBMSC. The CM from iBMSC/Vector or iBMSC/IL6 cells primed by LPC-CM were used for culturing with NB cells. **(G)** EdU incorporation assay. **(H)** Wound-healing assay. **(I)** The supernatant of iBMSC stimulated by LPC-CM was used for NB cells stimulation. (***p < 0.001 vs. iBMSC/Vector) Transwell migration assay was used to detect the migration ability of IMR32 and SK-N-AS cultured with hrIL6 supplemented into LPC-CM stimulated iBMSC supernatant. (**p < 0.01 and ***p < 0.001 vs. NC) Data represents three independent experiments.

LPC, as a natural product, has attracted widespread attention for its anti-tumor, antiviral, antibacterial and anti-inflammatory pharmacological properties. In order to evaluate its interfering effect on BMSC-NB cell interaction, NB cells pretreated with different doses of LPC were continued to be cultured in fresh medium for another 48, then the supernatant was extracted and as LPC pretreatment condition medium (LPC-CM). LPC-CM was used to prime the iBMSC, which were then co-cultured with non-LPC pretreated NB cells for a new round of culture. Thereafter, the tumor-promoting properties of NB cells-CM- and LPC–CM-priming iBMSC were observed. Interestingly, LPC-CM significantly affected the malignant transformation effect of iBMSC on NB cells in a dose dependent manner, which was manifested by the reduction in metastasis and proliferation of NB cells (Figures 4A–C). Meanwhile, we found that the secretion of IL6 in the co-culture system of LPC (especially 2.0 μM)-pretreated NB cells and iBMSC co-culture system was significantly downregulated compared with LPC untreated group (Figures 4D,E). Based on the above results, we speculated that IL6 secretion reduction might be responsible for the inhibition effect of LPC on NB cells-iBMSC interaction. Next, we found that overexpression of IL6 in iBMSC or supplement of hrIL6 into LPC-CM stimulated iBMSC supernatant significantly reversed the inhibitory effect of LPC on the pro-neoplastic of iBMSC (Figures 4F–I). The above suggested that LPC hinders the carcinogenic function of iBMSC by affecting IL6 production in iBMSC mediated by NB cells.

### Exogenous Overexpression of IL6 Reverses the Inhibitory Effect of Beta-Lapachone on Neuroblastoma Cell-Primed Cancer-Promoting Effect of Immortalized BMSC *in vivo*

**FIGURE 5:**
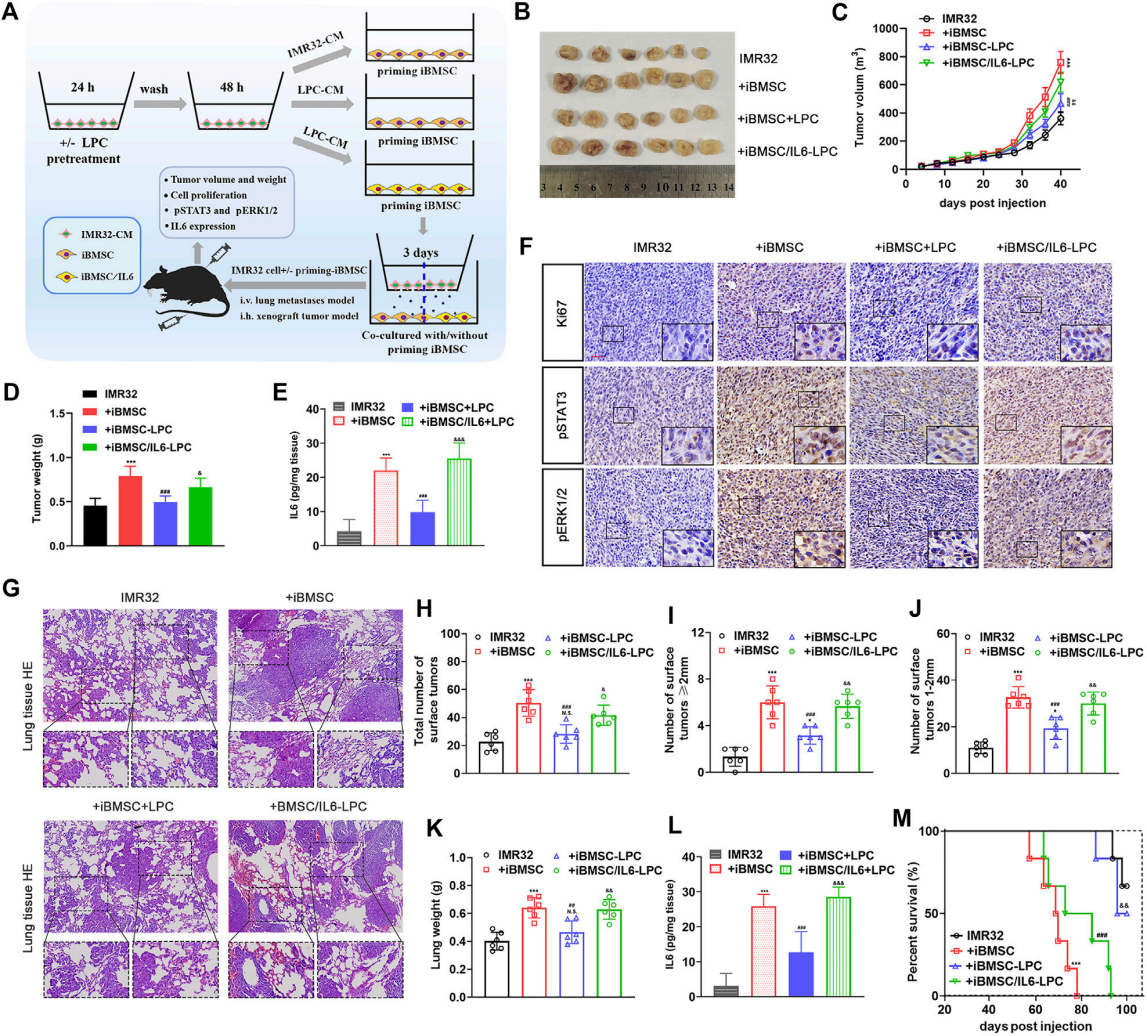
LPC suppresses the carcinogenic effect of IMR32-priming iBMSC *in vivo*, which could be reversed by exogenous overexpression of IL6. **(A)** The schematic diagram shows the preparation procedure of cells to be transplanted and the construction process of subcutaneous xenograft tumor and lung metastasis model in nude mice. Briefly, IMR32 was injected into the flanks of nude mice alone or mixed with IMR32-derived CM-priming iBMSC, LPC-CM-priming iBMSC or LPC-CM-priming IL6-expressing iBMSC (iBMSC/IL6), respectively. Meanwhile, the cells prepared in the same manner were intravenously injected into nude mice to establish the lung metastasis model. During modeling, another intravenous injection of iBMSC were performed at the second month of vaccination. **(B)** Representative images of tumor obtained from mice at 40 days after subcutaneous injection. **(C)** Tumor growth curve. **(D)** Tumor weight. **(E)** The expression level of IL6 in tumor tissues was determined by ELISA. **(F)** Representative images of IHC staining of Ki67, pSTAT3 and pERK1/2 in tumor tissues. **(G)** Representative HE staining images obtained from mouse lungs 2 months after tail vein injection. **(H–J)** The number of tumors on the lung surface. **(K)** Lung weight. **(L)** IL6 expression in lung metastatic lesions. **(M)** Survival curve of mice. (*p < 0.05, **p < 0.01 and ***p < 0.001 vs. IMR32; *#p* < 0.01, *##p* < 0.01 and *###p* < 0.001 vs. + iBMSC; *& p* < 0.05,*&& p* < 0.01 and *&&& p* < 0.0001 vs. + iBMSC + LPC).

To evaluate the interference of LPC on the cancer-promoting effect of iBMSC primed by NB cells *in vivo*, tumor xenotransplantation model and lung metastasis model were used. As shown in Figures 5A–F, IMR32 co-injected with iBMSC significantly promoted tumor growth and increased the number of KI67 + cells in tumor tissues. Interestingly, LPC-priming significantly inhibited the promoting effect of iBMSC on the proliferation and growth of IMR32-derived xenograft tumor. Furthermore, we found higher levels of IL6, pSTAT3 and pERK1/2 in tumor tissues in the IMR32 and BMSC co-injection group, which was significantly decreased upon LPC stimulation. However, overexpression of IL6 in iBMSC obviously alleviated the tumor suppression effect of LPC, which is manifested by the acceleration of tumor growth, the improvement of ki67 + cells and the up-regulation of pERK1/2 and pSTAT3 levels in tumor tissues. Subsequently, we observed relatively similar results in the IMR32-derived lung metastasis model as in the subcutaneous xenograft tumor model. As shown in Figures 5G–M, co-injection with iBMSC significantly improved the metastasis ability of IMR32, which was demonstrated by the lower survival time and rate of mice, the higher tumor burden, lung weight, and IL6 expression in lung metastatic lesions. Interestingly, LPC treatment significantly prolonged the survival time and rate of mice and reduced the number of lung tumor metastases and lesion occupancy, as well as IL6 expression in mice. However, iBMSC overexpressing IL6 was significantly resistant to LPC-CM stimulation. The above results altogether suggests that the anti-IL6 effect of LPC is responsible for its role in preventing iBMSC-mediated malignant transformation of NB cells *in vivo* and *in vitro*.

### Beta-Lapachone Disrupts the Gal-3/Gal-3BP/IL6 Signal Communication Between NB Cells and Immortalized BMSC in an NQO1-dependent Manner

**FIGURE 6:**
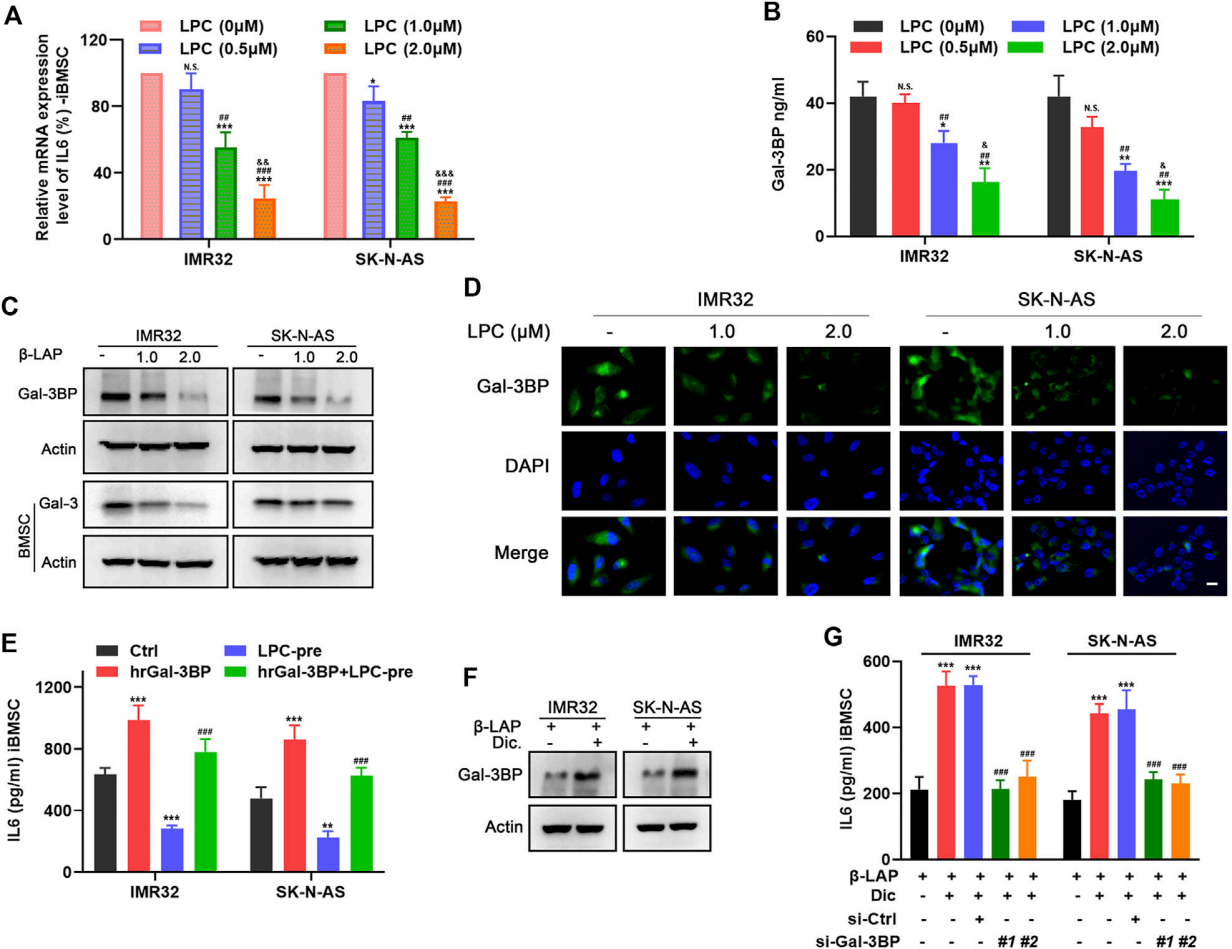
LPC suppresses iBMSC-derived IL6 production through inhibiting Gal-3/Gal-3BP signal in NQO1-dependent manner. LPC-CM obtained from NB cells treated with indicated doses of LPC was used for iBMSC culture. **(A)** The relative mRNA level of IL6 in iBMSC was detected by qPCR. **(B)** Gal-3BP secretion from NB cells CM was detected by ELISA. [*p < 0.05, **p < 0.01 and ***p < 0.001 vs. LPC (0 μM); *#p* < 0.01, *##p* < 0.01 and *###p* < 0.001 vs. LPC (0.5 μM); *& p* < 0.05,*&& p* < 0.01 and *&&& p* < 0.0001 vs. LPC (1.0 μM)] **(C)** The relative protein expression levels of Gal-3BP in NB cells and Gal-3 in iBMSC were detected by western blot. **(D)** The expression and localization of Gal-3BP in NB cells were detected by IF. **(E)** iBMSC was cultured with LPC-CM supplemented with or without hrGal-3BP, and the IL6 secretion was detected by ELISA. NB cells were pretreated with or without dicoumarin before LPC stimulation, and their CM was used to stimulate iBMSC. **(F)** The protein expression levels of Gal-3BP in NB cells were detected by western blot. (**p < 0.01 and ***p < 0.001 vs ctrl; *###p* < 0.001 vs LPC) **(G)** NB cells transfected with Gal-3BP siRNA or control siRNA were treated as shown in Figure 6G, and IL6 secretion in the iBMSC CM was detected by ELISA. (***p < 0.001 vs. LPC; *###p* < 0.001 vs. LPC + Dic) Data represents three independent experiments.

To determine the potential mechanism by which LPC interferes IL6 production in priming iBMSC, we examined the effects of LPC-CM on the mRNA expression and secretion of IL6 in iBMSCs. As shown in Figure 6A, LPC pretreatment significantly reduced the mRNA expression of IL6 in iBMSC mediated by IMR32 and SK-N-AS CM in a dose-dependent manner. As one of the important regulators of IL6 expression, Gal-3/Gal-3BP signal axis has been found to be widely expressed in a variety of NB cells and is involved in regulation of IL6 in iBMSCs. In order to investigate whether the inhibition of IL6 by LPC is related to the regulation of the Gal-3/Gal-3BP signal axis, we stimulated NB cells with increasing doses of LPC and the LPC-CM was extracted to stimulate the iBMSC. As shown in Figures 6B–D, the expression and secretion of Gal-3BP in NB cells was inhibited by LPC in a dose dependent manner. Meanwhile, we also explored the effect of LPC-CM on the expression of the receptor protein Gal-3 in iBMSC. We found that the expression of Gal-3 was decreased in iBMSC stimulated with the LPC-CM. When Gal-3BP was exogenously supplemented, we observed that LPC-induced inhibition of iBMSC-derived IL6 secretion was significantly reversed (Figure 6E). This indicates that the blocking effect of LPC on the secretion of Gal-3/Gal-3BP signal axis is partially responsible for weakening the interaction between NB cells and iBMSC. As the activator of LPC, NQO1 plays an important role in the pharmacological activity of LPC in cancer therapy. However, whether NQO1 acts as a meaningful response factor for LPC-mediated blockade of IL6 inflammatory signal remains to be explored. Interestingly, when the NQO1 inhibitor dicoumarin was administered, the suppressive effect of LPC on the Gal-3BP secretion in NB cells was significantly reversed (Figures 6F,G). In order to further clarify whether Gal-3BP was account for the IL6 inhibition effect of LPC activated by NQO1, two siRNAs targeting Gal-3BP with different sequences were transfected into NB cells in the presence or absence of dicoumarin, and then the IL6 secretion from iBMSC was detected. Knockdown efficiency of siRNA of Gal-3BP in NB cells was detected (Supplementary Figure S3). We found that dicoumarin pretreatment significantly reversed the IL6 stimulating activity of LPC-CM on iBMSC. Besides, the ability of iBMSCs to secrete IL6 in response to CM from Gal-3BP-silenced NB cells was not obviously restored even if dicoumarin was administered to inhibit LPC activity (Figures 6F,G). The above results indicated that LPC inhibits the synthesis and secretion of iBMSC-derived IL6 stimulated by NB cells in an NQO1-dependent manner, thus blocking the crosstalk between NB cells and iBMSC. This blocking effect was associated with the inhibition of the Gal-3BP paracrine effect of NB cells to iBMSC.

## Material and Methods

### Cell Culture

NB cell lines SK-N-BE (2), SK-N-SH and SH-SY5Y human BMSC were obtained from Cell Bank of the Chinese Academic of Sciences (Shanghai, China), SK-N-AS, IMR32 and BE (2) M17 were purchased from FuHeng biology (Shanghai, China). Human BMSC were obtained from Cell Bank of the Chinese Academic of Sciences (Shanghai, China), and the immortalized BMSC cell line (iBMSC) was established by ectopic expression of exogenous hTERT gene in human BMSC, as previously described (James et al., 2015), All cell lines were maintained according to the manufacturer’s instructions and cultured in a humidified incubator containing 5% CO2 at 37°C.

### Reagents

Beta-Lapachone (ARQ-501, LPC) was purchased from Selleck Chemicals Inc. RIPA buffer solution, TRIzol reagent, RIPA buffer solution and endotoxin-free plasmid small extraction medium volume extraction kit were obtained from Sparkjade Biotech (Shandong, China) AceQ qPCR SYBR Green Master Mix was purchased from Vazyme (Nanjing, China), and First-strand cDNA Synthesis super Mix kit was from TransGen Biotech (Beijing, China). HE staining kit was obtained from Beyotime Biotechnology (Hangzhou, China). EdU incorporation assay kit was obtained from Keygen Biotech (shanghai, China). Fetal Bovine Serum was purchased from Sigma-Aldrich (St. Louis, MO, United States). VECTASTAIN ABC Kit was purchased from Vector Laboratories. Human Galectin-3BP ELISA Kit was purchased from Abcam (Cambridge, MA, United States). Human IL6, IL6R ELISA kit were purchased from Boster Biological Technology (Nanjing, China). Primary antibodies we used were shown as following: ERK1/2 and *β*-Actin were purchased from abway. Gal-3, pSTAT3 and pERK1/2 were purchased from Cell Signaling Technology. Gal-3BP (LGALS3BP) was purchased from R&D Systems (Minneapolis, MN). STAT3 was purchased from Proteintech Group.

### Preparation of Conditioned Medium (CM)

According to the experimental requirements, NB cells and iBMSC were inoculated into the upper chamber and the lower chamber respectively and co-cultured for 24–48 h. Thereafter, the medium was changed to FBS-free medium, and the cells were cultured for another 48 h. NB cells or iBMSC cultured separately under the same conditions were used as controls. For the CM from NB cells treated with LPC or other reagents, the culture plate shall be cleaned with serum-free medium and replaced with new medium for another round of culture after administration. The medium was then collected and centrifuged at 1,000 × g at 4°C for 10 min and the supernatant was concentrated at 12,500 g for 30 min in a Microcon YM-3 centrifugal filter device (Millipore Co., Bedford, MA).

### *In vitro* IL6 Immuno-Suppression Assay

An anti-human IL-6 neutralizing antibody (AF206-NA) was used for IL6 suppression in CM derived from IMR32-priming iBMSC. Briefly, IMR32 was co-cultured with iBMSC for 24h, and then anti-IL-6 Abs (2.5 μg/ml) was administrated for another 24 h and the CM was extracted. Non-specific IgG antibody was used as a negative control (R&D Systems). Then, the CM above was used for stimulating the untreated IMR32 for 24–48 h. After that, the proliferation and migration of IMR32 were detected by clone formation assay and cell migration assay, respectively.

### Immunofluorescence Cell Staining

The cells in the logarithmic growth phase were digested with 0.25% trypsin, centrifuged and resuspended in fresh medium, and then seeded in a 24-well plate at a density of 10^4 cells per well and cultured for a specified time. After the cells were specifically treated, the corresponding antibodies were used to immunofluorescence stain the intracellular pERK1/2, pSTAT3 and Gal-3BP according to the operation instructions of the immunofluorescence kit.

### IHC and HE Staining

The IMR32-derived xenografts or patient-derived neuroblastoma tissues were fixed in 4% paraformaldehyde and then dehydrated with a gradient of alcohol, transparentized in xylene, embedded in paraffin, and cut to a thickness of 0.5 μm tissue section. IHC staining of indicated proteins in tumor tissue was performed using an IHC kit in accordance with the manufacturer’s instructions. The mouse lung tissue was removed and sectioned in the same manner as above. Tissue sections were stained using a HE staining kit according to manufacturer’s instructions. All sections were photographed under an upright microscope (Nikon, Japan).

### EdU Incorporation Assay

The cells were seeded into 96 well plates (5 × 10^3 cells per well) and incubated with EdU after indicated treatment. After incubation, the cells were fixed with 4% neutral paraformaldehyde and penetrated with 0.5% Triton X-100. Then click EdU reaction was carried out and photographed under inverted fluorescence microscope.

### Colony-formation Assay

The cells were stimulated by drugs or BMSC derived cm or specific treatment of other conditions before inoculation. Subsequently, these cells were inoculated into a six well plate (500 cells/well) for 2 weeks, during which the culture medium could be renewed 1–2 times. After that, the cells were fixed with 4% formaldehyde and stained with 0.1% crystal violet. Then, the number of clones is counted and images are collected.

### Total RNA Extraction and Real-Time Fluorescence Quantitative Polymerase Chain Reaction

Prepare RNase-free reagents and consumables in advance. TRIzol reagent is used to lyse the cell sample to be tested, and then extract total RNA. The absorbance of the extracted total RNA at 280 and 260 nm was detected by nanodrop spectrometer (Thermo Scientific) to evaluate its purity and concentration. Use the first-strand cDNA synthesis super-Mix kit to reverse transcribe qualified total RNA samples into cDNA. Real time quantitative PCR was performed in the light cycle 96 real-time PCR system (Roche) using aceq qPCR SYBR Green master mix. The mRNA expression level of IL6 was detected using a forward primer: 5′-ACA GCC ACT CAC CTC TTC AG-3′ and a reverse primer: 5′-CCA TCT TTT TCA GCC ATC TTT-3′, GAPDH was used as an internal control and was detected using forward primer: 5′-TGG TAT CGT GGA AGG ACT CA-3′ and reverse primer: 5′-CAG TAG AGG CAG GGA TGA TG-3’.

### Cell Viability Evaluation (MTT Assay)

Cell viability was detected as previously described (Zhou et al., 2019). Briefly, NB cells stimulated with or without CM from BMSC were inoculated on 96 well plates (3 × 10^3^ cells/well) and cultured for 12, 24, 48 and 72 h. After that, 0.5 mg/ml MTT (20 μL/well) was added and incubated for another 4 h. Cell viability was measured *via* the absorbance at 570 nm.

### Protein Extraction and Western Blot Analysis

The total protein was extracted using RIPA buffer and protein extraction kit according to the manufacturer’s instructions. After denaturation, SDS-PAGE gel electrophoresis, the protein sample was transferred to the membrane. Then the indicated primary and secondary antibodies were used to detect protein expression. All experimental procedures were carried out in accordance with standard manufacturer’s instructions.

### Enzyme Linked Immuno Sorbent Assay

Human IL6, IL6R and Gal-3BP ELISA kit (Boster Biological Technology) were used to determine the level of secretion or expression of specific proteins in cell lysates, culture supernatants, or tumor tissue, according to manufacturer’s instructions.

### Cell Migration Assay

Wound healing assay and Transwell migration assay were used to evaluate the migration ability of NB cells *in vitro*. For Wound healing assay: the cells were grown in a 96-well medium containing 10% FBS until the monolayer cell confluence reached about 70–80%. A sterile 200 µl spear head was then used to lightly scratch the cell layers to form equidistant gaps. After scratching, gently clean the plate holes with medium for 2 times to remove the exfoliated cells, and then add specific conditioned medium to continue culture. Cell migration was observed at different time points and images were taken under a microscope. For transwell migration assay: All cultured cells and iBMSC-derived CM were incubated at 37°C. NB cells in logarithmic growth phase were digested and re-suspended in complete medium and inoculated in upper chamber. The cells on the lower surface of the transwell chamber were then immersed in a 4% neutral paraformaldehyde solution and fixed, and stained with 0.1% crystal violet. The wound gap between the cells and the number of cells stained with crystal violet were measured using Photoshop and ImagJ software.

### Cell Transfection

The Gal-3BP-targeting siRNA were purchased from Qiagen (Valencia,CA). For transfection of siRNA, cells were inoculated into 24 well plates and cultured in antibiotic free medium before transfection until the cell growth confluence reached about 50–70%. Specific siRNA was transfected into the cells with lipo8000 transfection reagent (Beyotime Biotechnology) according to the manufacturer’s instructions. For the stable overexpression of IL6, lentivirus particles encoding IL6 and empty control vector (Han Biotechnology, Shanghai, China) were incubated with cells for 72 h, and cells infected with lentivirus particles were screened with purinmycin. Lentiviral particles encoding IL6 (Genomeditech, Shanghai, China) was incubated with cells for 72 h and cells infected with lentiviral particles were screened with puromycin.

### Xenograft Tumor and Lung Metastasis Mouse Model

The Balb/c nude mice purchased from Vitone River Laboratory Animal Technology Co., Ltd. (Beijing, China) were housed in a standard animal room with micro isolation devices. All the experimental methods of mice were approved by the animal ethics committee of Zhengzhou University (No. ZZUIRB 2021–99). Preparation of inoculated cell: IMR-32 pretreated with LPC were continued to be cultured in FBS-free medium for another 48 h, then the supernatant was extracted and concentrated as LPC pretreatment CM of IMR32 (LPC-CM). And iBMSC was primed with IMR32-derived CM (IMR32-CM) or LPC-CM. Then, IMR32 cultured alone or co-cultured with IMR32-CM-stimulated iBMSC or LPC-CM-stimulated iBMSC (with or without IL6 overexpressing) for another 3 days, and then they were collected and prepared for animal inoculation. Establishment of mouse tumor model: IMR-32 cell (1 × 10^6^ cells/mouse) was injected individually or co-injected with co-cultured iBMSC (ratio 1:1) into the subcutaneous or tail vein of mice to construct xenograft tumor model and lung metastasis model, respectively. For subcutaneously transplanted tumor-bearing mice: the subcutaneous tumor volume was measured every 4 days. The mice were euthanized on the 40 days after cell injection, then the tumor xenografts were removed and weighed. For IMR32-derived lung metastasis mouse model: During modeling, the corresponding iBMSC was reinjected into the mice *via* the caudal vein at second month, so as to replenish the iBMSC *in vivo*. Meanwhile, sterile PBS was injected as the control solvent. At the end of the experiment (2 months), the mice were euthanized and their lungs were removed and weighed. The size and number of tumor on the mouse lung surface were measured. The survival time of mice was recorded in the whole process of the survival experiment (100 days).

### Histological Sample

This study was approved by the ethics review committee of Zhengzhou University according to the standards and guidelines of the institutional review committee (No. 2021-H-K24). Untreated primary and BM metastatic tumors were obtained from NB patients with different risk stages. All patients were signed written informed consent in the Children’s Hospital Affiliated to Zhengzhou University. All studies on human samples were conducted in accordance with recognized ethical norms (Helsinki declaration, CIOMS, Belmont Report, American common rules).

### Statistical Analysis

All the results were obtained from at least three independent experiments and expressed as mean ± SD. Statistical analysis was performed with the *t*-test and one-way ANOVA (SPSS Software, Armonk, NY, United States) for two groups or multiple groups, respectively. Statistically significant difference was shown as *p < 0.05, **p < 0.01, and ***p < 0.001.

## Discussion

In recent decades, progress in effective treatment of NB has lagged far behind other childhood cancers, especially pediatric hematologic malignancies (Quinn et al., 2021). According to the International Neuroblastoma Risk Group (INRG) classification scheme, NB is classified as low, medium and high risk based on MYCN amplification status, metastasis, and whether the patient is older than 18 months (Monclair et al., 2009). At present, treatment options for high-risk NB patients are still limited, which may be partly due to the complex and polytropic tumor microenvironment (TME) (Borriello et al., 2016). The TME of NB consists of innate and adaptive immune cells, such as tumor-associated macrophages (TAM), dendritic cells, natural killer cells, and stromal cells, such as cancer-associated fibroblasts and BMSCs, as well as the chemokines and inflammatory cytokines secreted by these cells (Blavier et al., 2020). Therefore, it is necessary to deeply explore the characteristics and functions of TME in order to develop corresponding targeted new therapies.

An analysis of the MYCN non amplified NB subgroup of patients revealed higher TAM infiltration in high-risk metastatic NB tumors than in localized tumors. And the higher inflammation-related gene expression in metastatic NB tissues predicted the worse prognosis (Asgharzadeh et al., 2012). The expression level of IL6 in peripheral blood of untreated NB patients was found to be a potential indicator of disease extent and prognosis (Egler et al., 2008). Subsequently, rs1800795 single nucleotide polymorphism (SNP) of IL6 [-174 IL6 (G > C)] in NB samples was found to be an independent prognostic marker for high-risk NB (Lagmay et al., 2009). Notably, IL-6 has also been shown to function as a contributor to the dynamic crosstalk between tumour cells and the carcinoma-associated fibroblasts (CAFs) in the TME (Asgharzadeh et al., 2012). NB cells in BM metastases stimulate the secretion of BMSC-derived IL6, which can in turn act on NB cells through a paracrine effect (Ara et al., 2009). Thus it can be seen that IL6 may be an important link in the complexity of the cross-linking interaction between BMSC and NB cells.

However, the expression of IL6 in primary tumor tissues and BM metastases and its relationship with the disease feature of NB patients have not been fully elucidated. Here, we found that the expression level of IL6 was positively correlated with the risk level of patients both in primary and metastatic lesion of NB tissues. Bioinformatics analysis based on public databases also showed that high expression of IL6 gene was closely associated with poor prognosis of NB (GSE3960). However, given the high heterogeneity of cells in tumor tissues, it is not clear here whether IL6 is secreted from tumor cells, BMSC, TAM, CAFs or other types of cells in the TME of NB (Joshi, 2020). Nevertheless, our present results have provided strong evidence for the rationality of the inflammatory factor IL-6 as a prognostic risk assessment in NB. In addition, IL-6 produced by BM-derived suppressor cells, Dendritic cells and CAFs can also participate in the formation of tumor-promoting immunosuppression network (Tsukamoto et al., 2018). Contrarily, recent studies have reported the anti-tumor effect of IL6, which plays a key role in the activation, proliferation, and survival of lymphocytes during active immune responses by mobilizing anti-tumor T-cell immune responses (Fisher et al., 2014). Therefore, it is necessary to grasp the critical point between the pro-tumor and anti-tumor effects of IL6 in TME-based therapy, so as to carry out a correct and effective response.

LPC is a pleiotropic natural product, which has attracted much attention because of its strong antitumor activity in a variety of tumors (Gomes et al., 2021; Gong et al., 2021). LPC has limited therapeutic use because of its narrow treatment window, poor water solubility and high toxicity (Gomes et al., 2021). At present, a large number of studies focus on ameliorating the defects of LPC itself through formulation improvement or structural modification optimization, which makes it possible to regain clinical application (Blanco et al., 2007; Park et al., 2016; Jardim et al., 2017). LPC has played a role in inhibiting chronic inflammation in a variety of inflammatory diseases, such as acute pancreatitis, Alzheimer’s disease, multiple sclerosis, arthritis (Shen et al., 2017; Mokarizadeh et al., 2020). Although LPC exerts pharmacological activities in a variety of cancers and inflammatory diseases, its effect on the crosstalk between NB cells and the inflammatory TME has not been investigated. Using the IMR32-derived subcutaneous xenograft and lung metastasis mouse model, we had obtained evidence that LPC could effectively suppressed the promotion effect of iBMSC-derived IL6 on the growth and lung metastasis of NB tumor *in vivo*. Moreover, we found that LPC pretreatment hindered the promotion of IL6 gene expression and secretion of iBMSC induced by NB cells, which foreshadowed the antitumor effect of LPC against the inflammatory factor IL6. In our exploration of the mechanism of LPC, we found that NQO1 plays a decisive role in the efficacy of LPC. Based on the classic mechanism of LPC, we speculate that the inhibitory effect of IL6 may be related to the up-regulation of oxidative stress in NB cells mediated by LPC. However, this cannot completely rule out the effects of other products such as NADP+ and PARP-1 produced by the metabolism of LPC by NQO1 (Bey et al., 2013).

Gal-3BP is found to be a secretory protein associated with tumor malignancy and is expressed in most cancers (Giansanti et al., 2019). We and Yv et al. jointly demonstrated that Gal-3/Gal-3BP-mediated IL6 secretion may be one of the important factors in the malignant transformation of NB induced by BMSC (Silverman et al., 2012). They also detected the expression of Gal-3BP in most NB primary tissues collected, and found that Gal-3BP expression was higher in the samples of patients at stage 1–4 than that at stage 4S (metastasis and good prognosis), suggesting that Gal-3BP may indicate a poorer prognostic in patients. In addition, they also found IL6 expressing cells around Gal-3BP positive tumor cells in NB tissues, which reveals the close relationship between Gal-3BP expression and IL6 secretion. However, the changes of Gal-3BP secretion level in NB patients with different risk levels and the sequence of secretion changes between Gal-3BP and IL-6 have not been fully clarified.

Here, we detected the reduced secretion of Gal-3BP in NB cells and IL6 in iBMSC in the presence of LPC, which was reversed upon hrGal-3BP supplement, suggesting that IL6 secretion was significantly dependent on Gal-3BP. Importantly, NB cells-derived Gal-3BP was identified as a Gal-3-specific binding ligand, which can recognize and bind to Gal-3, and co-regulates transcriptional activation of IL6 in BMSC (Fukaya et al., 2008; Silverman et al., 2012). Therefore, Gal-3 plays an extremely important role in the Gal-3BP-mediated expression of IL6. In our study, it was found that LPC-CM also led to a slight down-regulation of Gal-3 expression in BMSC cells. Nevertheless, there is still a considerable amount of Gal-3 expression in LPC-CM-stimulated iBMSC. In view of this, we speculate that when Gal-3BP is replenished, the interaction of the intracellular residual Gal-3 with Gal-3BP may still partially restore the expression and secretion of IL6 in iBMSC. However, the importance of Gal-3 in this process needs to be further studied. These arguments will point to the new direction for future research on the effect of Gal-3/Gal-3BP/IL6 signaling on the TEM of NB.

In summary, our research provides new data support for the application of LPC in tumor inflammation related diseases, widens the scope of action of LPC based on tumor microenvironment, and provides a new research direction for the study of various pharmacological activities of LPC in the future.

## Conclusion

LPC pretreatment suppressed the iBMSC-mediated promotion of proliferation and metastases of NB cells through inhibition of IL6 secretion from iBMSC. Mechanistically, LPC disrupted the crosstalk between NB cells and iBMSC in an NQO1-dependent manner through blocking the Gal-3/Gal-3BP/IL6 axis, thereby inhibiting the carcinogenic effect of iBMSC-derived IL6 on NB cells.

## Data Availability

The original contributions presented in the study are included in the article/Supplementary Material, further inquiries can be directed to the corresponding authors.

## Abbreviations

BMSC, Bone marrow mesenchymal stem cell; iBMSC, immortalized BMSC; CM, Conditioned medium; Gal-3, Galectin-3; Gal-3BP, Galectin-3-binding protein; IL6, Interleukin 6; LPC, Beta-Lapachone; NB, Neuroblastoma; NQO1, NAD(P)H quinone dehydrogenase 1; TME, Tumor microenvironment.
