# Supplementary material for: Beta-Lapachone Attenuates BMSC-Mediated Neuroblastoma Malignant Transformation by Inhibiting Gal-3/Gal-3BP/IL6 Axis
Source: Front Pharmacol. 2021 Nov 1;12:766909. doi: 10.3389/fphar.2021.766909 (PMC8591123; doi:10.3389/fphar.2021.766909)
Supplement: Supplementary file 1 [file DataSheet1.docx]

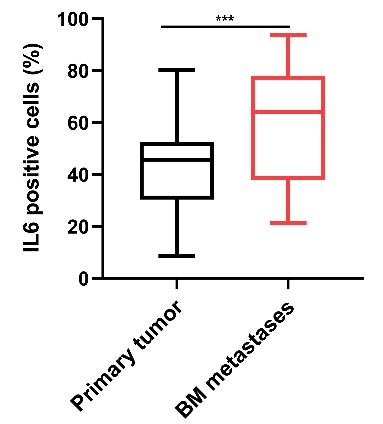


Supplementary Figure 1.

The expression difference of IL6 between NB primary tissues and BM metastases. ***P<0.001 compared with NB primary tissues.


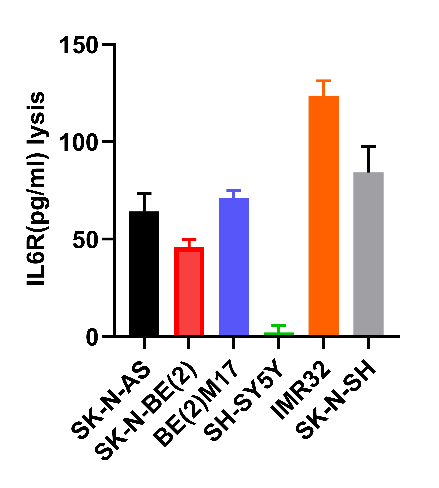


Supplementary Figure 2. Six NB cell lines were cultured under normal conditions. The expression of IL6R in the protein lysate of NB cells was detected by ELISA. Data represents three independent experiments and is presented as mean ± SD


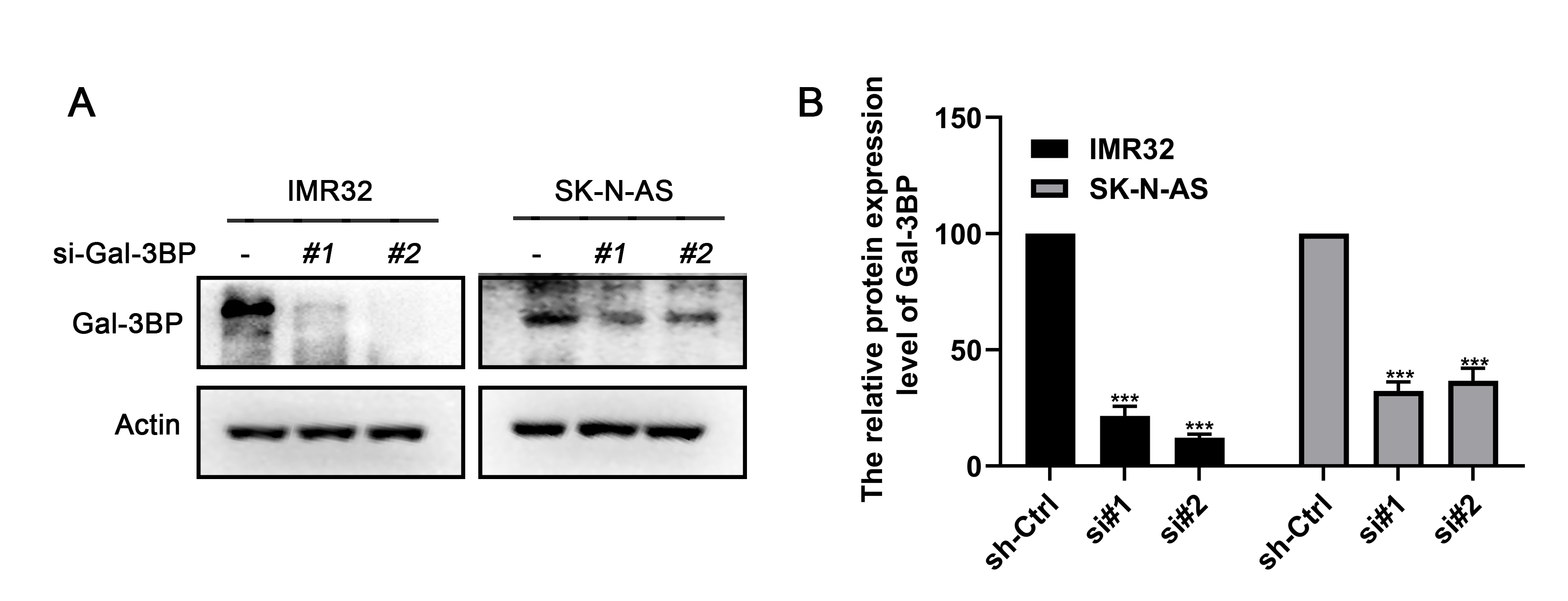


Supplementary Figure 3. Knockdown efficiency of siRNA of Gal-3BP in NB cells. Two siRNAs targeting Gal-3BP were transfected into SK-N-AS and IMR32 cells. (A) Western blot was used to detect the expression of Gal-3BP. (B) Corresponding quantization results. ***P<0.001 compared with shCtrl group. Data represents three independent experiments.
